# Teachers' self-efficacy and its relationship to inclusive education for students with autism: a cross-sectional study

**DOI:** 10.3389/fpsyg.2026.1811958

**Published:** 2026-08-11

**Authors:** Mohammed Abdulrhman Alassaf

**Affiliations:** Department of Special Education, College of Education, Imam Mohammad Ibn Saud Islamic University (IMSIU), Riyadh, Saudi Arabia

**Keywords:** autism spectrum disorder, inclusive education, Saudi Arabia, self-efficacy, teachers

## Abstract

**Background:**

Inclusive education is increasingly recognized as an important approach to promoting equitable learning opportunities for all students, including students with autism spectrum disorder (ASD). Teachers' self-efficacy, or their perceived capability to manage classrooms, adapt instruction, and support diverse learners, is considered an important factor in autism inclusion. However, limited research in Saudi Arabia has examined teachers' self-efficacy in relation to self-reported inclusive education orientations toward students with ASD.

**Objective:**

This study aimed to assess teachers' self-efficacy and self-reported inclusive education orientations toward students with ASD in Riyadh, Saudi Arabia. It also examined whether selected demographic and professional characteristics were associated with self-efficacy and inclusive education orientations.

**Methods:**

A cross-sectional correlational design was used. A convenience sample of 381 general and special education teachers from mainstream schools in Riyadh participated in the study. Data were collected using a structured, self-administered questionnaire covering demographic and professional characteristics, teachers' self-efficacy, and inclusive education orientations toward students with ASD. Descriptive statistics, independent-samples *t*-tests, one-way ANOVA, Pearson's correlation, and multiple linear regression were conducted using SPSS version 27.

**Results:**

Teachers reported moderately high levels of self-efficacy (*M* = 3.84, *SD* = 0.62) and generally positive inclusive education orientations (*M* = 3.88, *SD* = 0.59). A strong positive correlation was found between self-efficacy and inclusive education orientations (*r* =0.62, *p* < 0.001). In the regression model, self-efficacy showed the strongest association with inclusive education orientations (β =0.48, *p* < 0.001). Prior training on inclusion, longer teaching experience, special education teaching role, and prior experience teaching students with ASD were also significantly associated with more positive inclusive education orientations.

**Conclusion:**

The findings indicate that teachers' self-efficacy is closely associated with self-reported inclusive education orientations toward students with ASD among the participating teachers. They also suggest that targeted training, teaching experience, teaching role, and autism-specific exposure may be relevant factors in supporting inclusive education. Because of the cross-sectional design, convenience sampling, and reliance on self-report data, the findings should be interpreted as evidence of association rather than causation, population-level generalizability, or direct evidence of observed classroom practice.

## Introduction

Inclusive education has become a major priority in contemporary educational reform, reflecting the principle that all students, including students with autism spectrum disorder (ASD), should have equitable access to meaningful learning opportunities within mainstream school settings (Avramidis and Norwich, 2002). However, the success of inclusion depends not only on policy commitments, but also on teachers' confidence, preparation, and perceived ability to respond to diverse learning needs (Sharma et al., 2012). This issue is particularly important in autism inclusion because students with ASD may require support in communication, social interaction, sensory regulation, classroom participation, behavior management, and individualized learning adaptation (Wittwer et al., 2024). Therefore, teachers' beliefs about their own capability to support autistic students represent an important psychological and professional factor in inclusive education (Woodcock and Jones, 2020).

Teacher self-efficacy is a central construct in understanding how teachers approach inclusion (Herzig Johnson, 2023). Rooted in Bandura's social cognitive theory, self-efficacy refers to individuals' beliefs in their capability to organize and execute the actions required to manage prospective situations (Bandura and Wessels, 1997). In educational contexts, these beliefs influence how teachers respond to instructional challenges, persist when difficulties arise, adapt teaching strategies, and manage classroom demands (Waddington, 2023). Teachers with stronger self-efficacy are generally more likely to report confidence in using flexible instructional approaches, supporting student engagement, collaborating with others, and responding constructively to diverse classroom needs (Woodcock and Jones, 2020).

Bandura's framework is especially relevant to inclusive education because self-efficacy is not a fixed personal trait, but a dynamic belief shaped by experience, professional preparation, and contextual support (Bandura and Wessels, 1997). One of the most important sources of self-efficacy is mastery experience, meaning that confidence may develop through repeated successful engagement with teaching tasks (Waddington, 2023). At the same time, stronger self-efficacy may encourage teachers to persist, experiment with new strategies, and engage more positively with complex classroom situations (Herzig Johnson, 2023). This suggests a potentially reciprocal relationship: successful inclusive experiences may strengthen teachers' self-efficacy, while higher self-efficacy may support more positive orientations toward inclusion (Woodcock and Jones, 2020). Because the present study used a cross-sectional design, this relationship is examined as an association rather than as a causal pathway.

The literature on inclusive education has consistently shown that teachers' beliefs, attitudes, preparation, and perceived competence are important to the implementation of inclusion (Avramidis and Norwich, 2002). Avramidis and Norwich (2002) showed that teachers' attitudes toward inclusion are influenced by factors such as training, experience, and the nature of students' needs, while also emphasizing that inclusion is shaped by broader contextual and structural conditions. Similarly, Sharma et al. (2012) highlighted the importance of teacher efficacy for inclusive practice, particularly in relation to inclusive instruction, collaboration, and behavior management (Friesen et al., 2023). These studies indicate that inclusive education is not determined by willingness alone, but by the interaction of professional confidence, preparation, classroom demands, and institutional support (Sharma et al., 2012).

Autism inclusion requires particular attention because the educational needs of autistic students may differ from those of other students with disabilities (Youssif et al., 2024). Students with ASD may experience challenges related to expressive and receptive communication, peer interaction, transitions, restricted interests, sensory sensitivities, emotional regulation, and participation in group-based classroom activities (Wittwer et al., 2024). These characteristics can require teachers to use structured routines, individualized supports, visual strategies, differentiated instruction, collaborative planning, and positive behavior support (Bowman and Harrison, 2025). As a result, teacher self-efficacy in autism inclusion is not only a general belief about teaching ability, but a context-specific belief about the capacity to respond to autism-related instructional, behavioral, social, and communication needs (Youssif et al., 2024).

Previous research supports this autism-specific perspective (Wittwer et al., 2024). Wittwer et al. (2024) found that teachers' autism-related knowledge predicted their confidence and attitudes toward including autistic students. Bowman and Harrison (2025) reported that higher autism knowledge among pre-service teachers was associated with stronger self-efficacy, which in turn mediated attitudes toward inclusion. Youssif et al. (2024) also identified differences in self-efficacy between teachers working with students with intellectual disabilities and those teaching students with ASD, suggesting that autism inclusion may require particular forms of professional competence and preparation (Guo et al., 2021; Monteiro and Forlin, 2023). These findings suggest that teachers' confidence in autism-inclusive classrooms is shaped not only by general teaching experience, but also by autism-specific knowledge, training, and exposure (Bowman and Harrison, 2025).

Despite this growing body of work, important gaps remain in understanding how teacher self-efficacy relates to autism-specific inclusion across different educational contexts (Al-Dakroury et al., 2022). Much of the literature reports associations between training, experience, self-efficacy, and inclusion-related outcomes, but the mechanisms connecting these factors are not always clearly explained (Martins and Chacon, 2021). Training may strengthen teachers' confidence by improving autism-related knowledge, modeling effective strategies, reducing uncertainty, and increasing familiarity with inclusive classroom adaptations (Johnson et al., 2021). Similarly, teaching experience may strengthen self-efficacy through repeated mastery experiences and greater exposure to diverse learners (Waddington, 2023). However, these relationships may also depend on school-level conditions, including availability of resources, support staff, administrative leadership, collaboration structures, and inclusive school culture (Avramidis and Norwich, 2002). Therefore, teachers' self-efficacy should be examined not as an isolated personal characteristic, but as a professional belief shaped by both individual preparation and educational context (Sharma et al., 2012).

This issue is especially relevant in Saudi Arabia, where autism services and inclusive education have expanded in recent years, but challenges remain in teacher preparation, school-based support, and service development (Al-Dakroury et al., 2022). Al-Dakroury et al. (2022) noted that autism-related services and research in the Kingdom of Saudi Arabia continue to face developmental gaps despite increasing recognition of ASD. Within mainstream school settings, these challenges may influence how confident teachers feel in supporting autistic students and how positively they report their orientation toward inclusion (Alhumaid, 2021). Existing Saudi research has begun to highlight this issue, particularly in relation to teacher self-efficacy and inclusion of students with autism (Alhumaid, 2021). For example, Alhumaid (2021) found that teacher self-efficacy was relevant to the inclusion of students with autism in Saudi educational settings, while also identifying contextual barriers that may affect implementation. Nevertheless, empirical studies examining teachers' self-efficacy and autism-specific inclusive education orientations in Saudi mainstream schools remain limited (Al-Dakroury et al., 2022).

Accordingly, the present study was designed to examine teachers' self-efficacy and its association with self-reported inclusive education orientations toward students with ASD in Riyadh, Saudi Arabia. In this study, inclusive education orientations refer to teachers' reported attitudes, beliefs, contextual perceptions, and selected reported inclusion-related behaviors, rather than directly observed classroom practice. This distinction is important because the study relied on a self-administered questionnaire and did not include classroom observation. By focusing on the Saudi mainstream school context, the study seeks to contribute local evidence to the broader literature on autism inclusion and clarify how teachers' perceived capability relates to their reported orientation toward inclusive education.

Based on the reviewed literature and the identified gaps, this study was guided by the following research questions:

1. What is the level of teachers' self-efficacy in supporting inclusive education for students with ASD in Riyadh, Saudi Arabia?
2. What is the level of teachers' self-reported inclusive education orientations toward students with ASD?
3. Do teachers' self-efficacy scores differ according to selected demographic and professional characteristics?
4. To what extent is teachers' self-efficacy associated with inclusive education orientations after accounting for selected demographic and professional factors?

By addressing these questions, the study aims to provide context-specific evidence on the role of teacher self-efficacy in autism inclusion within Saudi mainstream schools.

## Methodology

### Research design

This study used a cross-sectional correlational design to examine the association between teachers' self-efficacy and self-reported inclusive education orientations toward students with autism spectrum disorder (ASD) in mainstream schools in Riyadh, Saudi Arabia. Data were collected at a single point in time using a structured questionnaire. This design was appropriate for describing teachers' perceived capabilities, beliefs, and reported inclusion-related behaviors, and for examining associations among these variables within a natural educational setting.

Because the study was cross-sectional, it did not permit conclusions about causality or the temporal direction of relationships between variables. Therefore, all findings were interpreted as associations rather than causal effects. In addition, the study measured self-reported perceptions and reported practices, rather than directly observed classroom practice.

### Research population

The target population comprised teachers working in mainstream schools in Riyadh, Saudi Arabia, who were involved in teaching or supporting students with ASD in inclusive educational settings. This included both general education teachers and special education teachers. General education teachers are directly involved in classroom instruction and day-to-day inclusion in mainstream classrooms, whereas special education teachers provide more specialized instructional, behavioral, and consultative support for students with ASD. Teachers from primary, intermediate, and secondary school levels were included to capture perspectives across educational stages.

An official population count for all general and special education teachers involved in autism inclusion in mainstream schools in Riyadh was not available. The only accessible official figure obtained from the Ministry of Education was approximately 3,905 special education teachers in Riyadh. This figure was therefore used as the reference population for sample size estimation. However, recruitment was intentionally extended to both general education and special education teachers because both groups contribute meaningfully to the implementation of inclusive education for students with ASD.

### Research sample and sampling strategy

The final sample consisted of 381 teachers working in mainstream schools in Riyadh, Saudi Arabia. Participants included both general education teachers and special education teachers because autism inclusion in mainstream schools depends on the contribution of both classroom-based instructional roles and specialized support roles.

Because no complete official sampling frame was available for all general and special education teachers involved in autism inclusion in mainstream schools in Riyadh, a formal probability-based sample size calculation for the full target population could not be conducted. The only accessible official figure obtained from the Ministry of Education was approximately 3,905 special education teachers in Riyadh. This figure was therefore used only as a pragmatic reference to guide recruitment planning, not as the basis for claiming a fixed confidence interval or margin of error for the mixed sample of general and special education teachers.

Participants were recruited using convenience sampling. Recruitment was conducted through schools and school communication channels and was open to eligible teachers who were available during the data collection period. The use of non-probability convenience sampling may limit representativeness and generalizability. Therefore, the findings should be interpreted as reflecting the participating teachers rather than all teachers in Riyadh or Saudi Arabia.

Although the sample size was considered adequate for the planned descriptive, group comparison, correlational, and regression analyses, the absence of a complete sampling frame and the inclusion of both general and special education teachers mean that the sample should not be interpreted as statistically representative of the full teacher population involved in autism inclusion.

### Data collection instrument

Data were collected using a structured, self-administered questionnaire designed to assess teachers' self-efficacy and their self-reported inclusive education attitudes, beliefs, contextual perceptions, and selected reported practices toward students with ASD. The questionnaire consisted of three sections:

A) demographic and professional characteristics,
B) teachers' self-efficacy, and
C) inclusive education orientations toward students with ASD.

The term “inclusive education orientations” was used to reflect that the outcome section included conceptually related but not identical components, including attitudes toward inclusion, reported inclusion-related behaviors, perceived training importance, collaboration, and perceived school resources. Because the study relied on self-report data, the outcome should be understood as teachers' reported orientations toward inclusive education rather than directly observed inclusive classroom practice.

To enhance accessibility and comprehension, the questionnaire was prepared in both English and Arabic. Based on participant preference, 286 teachers completed the Arabic version and 95 completed the English version. The Arabic version was produced using translation and back-translation procedures and was reviewed by bilingual experts to ensure linguistic accuracy, conceptual equivalence, and cultural appropriateness.

The full questionnaire was also evaluated by a panel of seven experts in psychology, educational psychology, special education, autism, psychometrics, curriculum and instruction, and inclusive education. Expert feedback was used to refine item wording, improve clarity, and strengthen relevance to the Saudi educational context. The revised instrument was then pilot-tested with 30 teachers who were not included in the final sample. The pilot test assessed item clarity, comprehensibility, and completion time. Only minor linguistic and formatting modifications were required before full administration.

### Section A: demographic and professional characteristics

This section collected background information about participants, including gender, age, educational qualification, years of teaching experience, teaching role, school level, previous training related to inclusive education, and prior experience teaching students with ASD. These variables were used to describe the sample, compare relevant teacher groups, and examine their associations with self-efficacy and inclusive education orientations.

### Section B: teachers' self-efficacy

Teachers' self-efficacy was measured using eight items adapted from the long form of the Teachers' Sense of Efficacy Scale. The items were contextualized to autism inclusion and focused on teachers' perceived capability to perform inclusion-related teaching tasks. These tasks included managing classroom behavior, adapting instruction, creating a supportive learning environment, collaborating with staff, assessing student progress, motivating students with ASD, and managing teaching-related stress.

Responses were rated on a 5-point Likert scale ranging from 1 = strongly disagree to 5 = strongly agree. Higher scores indicated stronger perceived self-efficacy. For analysis, the self-efficacy score was calculated as the mean of the eight items.

This construct was conceptually defined as teachers' beliefs about their own capability to organize and perform autism-inclusive teaching tasks. Therefore, it differs from the outcome construct, which assessed teachers' reported attitudes, beliefs, contextual perceptions, and selected reported inclusion-related behaviors.

### Section C: inclusive education orientations toward students with ASD

The third section included eight items assessing teachers' self-reported orientations toward inclusive education for students with ASD in mainstream classrooms. These items addressed perceived benefits of inclusion, support for mainstream placement where appropriate, reported adaptation of classroom materials, openness to learning new strategies, perceived importance of autism-related teacher training, collaboration with parents, perceived adequacy of school resources, and perceived social benefits of inclusion.

Responses were rated on the same 5-point Likert scale ranging from 1 = strongly disagree to 5 = strongly agree. Higher scores indicated more positive inclusive education orientations. The total score was calculated as the mean of the eight items.

Because the items included attitudes, beliefs, contextual perceptions, and selected reported behaviors, the scale was not interpreted as a pure measure of actual inclusive practice. Instead, it was treated as a self-reported inclusive education orientation scale. This distinction was important because the study did not include classroom observation or external assessment of teaching practice.

### Conceptual distinction between the two main constructs

Although teachers' self-efficacy and inclusive education orientations are theoretically related, they were treated as distinct constructs in this study. Self-efficacy refers to teachers' perceived capability to perform specific autism-inclusive teaching tasks. In contrast, inclusive education orientations refer to teachers' reported attitudes, beliefs, contextual perceptions, and selected reported behaviors related to including students with ASD in mainstream classrooms.

The distinction is important because a teacher may feel capable of adapting instruction or managing classroom behavior, but may still hold different beliefs about inclusion, perceive inadequate school resources, or vary in reported collaboration and implementation. Therefore, self-efficacy was treated as the main explanatory variable, whereas inclusive education orientations were treated as the outcome variable. However, because both constructs were measured using self-report Likert-scale items, the possibility of shared method variance and conceptual proximity was acknowledged and considered when interpreting the correlation and regression findings.

### Content validity and psychometric evaluation

Content validity was addressed through expert review, translation and back-translation, pilot testing, and item refinement. The expert panel assessed whether the items were clear, relevant, and appropriate for measuring teacher self-efficacy and inclusive education orientations in the context of autism inclusion in Saudi mainstream schools.

Internal consistency reliability was assessed using Cronbach's alpha. The teachers' self-efficacy scale showed good internal consistency, with a Cronbach's alpha of 0.88. The inclusive education orientation scale also demonstrated good internal consistency, with a Cronbach's alpha of 0.85. Corrected item-total correlations were examined to ensure that all items contributed meaningfully to their respective scales. Corrected item-total correlations ranged from 0.56 to 0.74 for the self-efficacy scale and from 0.52 to 0.71 for the inclusive education orientation scale.

Exploratory factor analysis was conducted to examine the internal structure of the questionnaire. The suitability of the data for factor analysis was confirmed using the Kaiser-Meyer-Olkin measure of sampling adequacy and Bartlett's test of sphericity. For the self-efficacy scale, the KMO value was 0.89, and Bartlett's test of sphericity was statistically significant, χ^2^(28) = 1374.62, *p* < 0.001. For the inclusive education orientation scale, the KMO value was 0.86, and Bartlett's test was also statistically significant, χ^2^(28) = 1198.45, *p* < 0.001.

Principal axis factoring was used as the extraction method because the aim was to identify the underlying latent structure of the scales. Each scale was examined separately because the two constructs were theoretically distinct. Since each scale showed a dominant single-factor solution, rotation was not applied. The self-efficacy scale produced a one-factor solution with an eigenvalue of 4.36, explaining 54.5% of the variance. Factor loadings ranged from 0.64 to 0.81. The inclusive education orientation scale also produced a one-factor solution with an eigenvalue of 4.08, explaining 51.0% of the variance. Factor loadings ranged from 0.60 to 0.79.

No items showed weak conceptual relevance or problematic loading patterns that required removal. Cross-loadings were not examined because each scale was analyzed separately as a theoretically defined scale. These findings provided preliminary support for the internal consistency and construct validity of the adapted questionnaire in the present sample. However, because the questionnaire was administered in both Arabic and English, and formal measurement invariance testing between the two language versions was not conducted, linguistic equivalence should be interpreted cautiously. Future studies should conduct confirmatory factor analysis and measurement invariance testing to further evaluate the psychometric performance of the instrument across language groups.

### Data collection procedure

Data were collected from teachers working in mainstream schools in Riyadh, Saudi Arabia. The questionnaire was distributed in both electronic and paper-based formats to improve accessibility and participation. Prior to data collection, permission to approach schools was obtained through the relevant administrative channels. School principals were contacted to facilitate access to eligible participants.

Teachers who met the inclusion criteria were invited to participate voluntarily. The questionnaire was available in both Arabic and English, and participants completed their preferred language version. Recruitment took place through direct distribution during school meetings and through electronic circulation via email and official school communication channels.

Before completing the questionnaire, participants received an informed consent statement explaining the purpose of the study, the voluntary nature of participation, the right to withdraw at any time, and assurances regarding anonymity and confidentiality. Participants were given approximately 2 weeks to complete the questionnaire, and follow-up reminders were issued during the data collection period to encourage participation and reduce non-response.

After data collection, completed questionnaires were checked for completeness and coded for analysis. Electronic responses were downloaded into a protected database, and paper-based responses were entered manually into the dataset. All data were stored securely and accessed only for research purposes.

## Data analysis

Data were analyzed using SPSS version 27. Prior to analysis, the dataset was screened for completeness, coding accuracy, and potential entry errors. Descriptive statistics, including frequencies, percentages, means, and standard deviations, were used to summarize demographic and professional characteristics and the main study variables.

Internal consistency reliability was examined using Cronbach's alpha coefficients. Construct validity was assessed using exploratory factor analysis. Principal axis factoring was used to examine the internal structure of the teachers' self-efficacy scale and the inclusive education orientation scale. Harman's single-factor test was also conducted using all self-efficacy and inclusive education orientation items to examine the potential influence of common method variance. Scale scores for teachers' self-efficacy and inclusive education orientations were computed as the mean of the relevant items, with higher scores indicating higher levels of the corresponding construct.

Independent-samples *t*-tests and one-way analysis of variance were used to examine differences in teachers' self-efficacy across selected demographic and professional characteristics, including gender, educational qualification, teaching experience, previous training on inclusion, teaching role, school level, and prior experience teaching students with ASD. Additional group comparisons were conducted to examine differences in inclusive education orientation scores according to teaching role and prior experience teaching students with ASD. Where ANOVA results were statistically significant, *post hoc* comparisons were conducted to identify the location of group differences. Effect sizes were considered in interpretation, including Cohen's d for *t*-tests and eta squared for ANOVA. Because multiple group comparisons were conducted, the Benjamini–Hochberg false discovery rate procedure was applied to reduce the risk of type I error while maintaining appropriate sensitivity for exploratory comparisons.

Pearson's correlation coefficient was used to examine the association between teachers' self-efficacy and inclusive education orientations. Multiple linear regression analysis was then conducted to examine whether self-efficacy remained associated with inclusive education orientations after adjusting for selected demographic and professional variables.

In response to the conceptual relevance of teacher role and autism-specific experience, the regression model included the following predictors: teachers' self-efficacy, gender, teaching experience, previous training on inclusion, educational qualification, teaching role, school level, and prior experience teaching students with ASD. Categorical variables were coded as follows: gender was coded as male = 0 and female = 1; previous training on inclusion was coded as no = 0 and yes = 1; teaching role was coded as general education teacher = 0 and special education teacher = 1; and prior experience teaching students with ASD was coded as no = 0 and yes = 1. Educational qualification, teaching experience, and school level were entered using dummy coding. Diploma qualification, less than 5 years of teaching experience, and primary school level were used as the reference categories.

Regression results were reported using unstandardized coefficients, standard errors, standardized beta coefficients, 95% confidence intervals, *t*-values, and *p*-values. Model fit was assessed using *R*^2^, adjusted *R*^2^, F-statistic, and model significance. Regression assumptions were examined before interpretation, including linearity, independence of errors, homoscedasticity, normality of residuals, absence of problematic outliers, and multicollinearity. Multicollinearity was assessed using tolerance and variance inflation factor values. Tolerance values ranged from 0.71 to 0.94, and VIF values ranged from 1.06 to 1.41, indicating no problematic multicollinearity among the predictors. The Durbin-Watson statistic was 1.91, supporting the independence of residuals.

A significance level of *p* < 0.05 was adopted for all statistical tests. Because several inferential tests were performed, findings were interpreted cautiously, with attention to effect sizes, adjusted significance patterns, and theoretical relevance rather than statistical significance alone.

Because the study used self-report questionnaire data, the results were interpreted as reflecting teachers' perceived self-efficacy and reported inclusive education orientations rather than directly observed inclusive classroom practice. Responses from teachers without prior direct experience teaching students with ASD were retained because the instrument assessed perceived capability and orientations toward inclusion; however, prior ASD teaching experience was examined as a relevant professional variable.

## Results

## Sample description

A total of 381 teachers participated in the study. As shown in Table 1, 213 participants were female (55.9%) and 168 were male (44.1%). The largest age group was 30–39 years (*n* = 128, 33.6%), followed by 40–49 years (*n* = 101, 26.5%), younger than 30 years (*n* = 92, 24.1%), and 50 years or older (*n* = 60, 15.8%).

**Table 1:** Sample characteristics of the participants (*N* = 381).

Variable	Category	*n*	%
Gender	Male	168	44.1
	Female	213	55.9
Age	< 30 years	92	24.1
	30–39 years	128	33.6
	40–49 years	101	26.5
	≥50 years	60	15.8
Educational qualification	Diploma	47	12.3
	Bachelor's	211	55.4
	Master's	94	24.7
	PhD	29	7.6
Teaching experience	< 5 years	88	23.1
	5–10 years	134	35.2
	11–15 years	96	25.2
	>15 years	63	16.5
Type of teacher	General education	232	60.9
	Special education	149	39.1
School level	Primary	145	38.1
	Intermediate	121	31.8
	Secondary	115	30.2
Previous training on inclusive education	Yes	176	46.2
	No	205	53.8
Experience teaching students with autism	Yes	192	50.4
	No	189	49.6

Regarding educational qualification, most participants held a bachelor's degree (*n* = 211, 55.4%), followed by a master's degree (*n* = 94, 24.7%), diploma (*n* = 47, 12.3%), and PhD (*n* = 29, 7.6%). In terms of teaching experience, the largest proportion had 5–10 years of experience (*n* = 134, 35.2%), followed by 11–15 years (*n* = 96, 25.2%), less than 5 years (*n* = 88, 23.1%), and more than 15 years (*n* = 63, 16.5%).

Most participants were general education teachers (*n* = 232, 60.9%), while 149 participants were special education teachers (39.1%). With respect to school level, 145 participants worked in primary schools (38.1%), 121 in intermediate schools (31.8%), and 115 in secondary schools (30.2%). In addition, 176 teachers (46.2%) reported previous training on inclusive education, whereas 205 teachers (53.8%) had not received such training. Nearly half of the sample had prior experience teaching students with autism (*n* = 192, 50.4%), while 189 participants (49.6%) reported no such experience.

## Psychometric evaluation of the study scales

The teachers' self-efficacy scale demonstrated good internal consistency reliability, with a Cronbach's alpha of 0.88 and corrected item–total correlations ranging from 0.56 to 0.74. The data were suitable for factor analysis, as indicated by a Kaiser–Meyer–Olkin (KMO) measure of 0.89 and a significant Bartlett's test of sphericity, χ^2^(28) = 1374.62, *p* < 0.001. Principal axis factoring supported a unidimensional structure, with the first factor yielding an eigenvalue of 4.36 and accounting for 54.5% of the total variance. Factor loadings ranged from 0.64 to 0.81. The remaining eigenvalues were all below 1.00 (0.82, 0.71, 0.59, 0.48, 0.39, 0.35, and 0.30), further supporting retention of a single-factor solution (Table 2).

**Table 2:** Psychometric properties and factor-analytic results of the study scales.

Analysis/scale	Cronbach's α	Corrected item-total correlatio*n* range	KMO	Bartlett's test	Eigenvalue series	Variance explained by first Factor	Factor loading Range
Teachers' self-efficacy	0.88	0.56–0.74	0.89	χ^2^(28) = 1374.62, *p* < 0.001	4.36, 0.82, 0.71, 0.59, 0.48, 0.39, 0.35, 0.30	54.5%	0.64–0.81
Inclusive education orientations	0.85	0.52–0.71	0.86	χ^2^(28) = 1198.45, *p* < 0.001	4.08, 0.86, 0.74, 0.63, 0.51, 0.44, 0.39, 0.35	51.0%	0.60–0.79
Harman's single-factor test	-	-	0.91	χ^2^(120) = 3,486.72, *p* < 0.001	6.19, 1.48, 1.12, 0.94, 0.83, 0.74, 0.68, 0.61, 0.55, 0.49, 0.43, 0.38, 0.33, 0.26, 0.22, 0.15	38.7%	-

KMO = Kaiser–Meyer–Olkin measure of sampling adequacy; χ^2^ = Bartlett's test of sphericity; factor extraction method = principal axis factoring. Harman's single-factor test was conducted using all 16 items from the teachers' self-efficacy and inclusive education orientation scales. The first unrotated factor explained 38.7% of the total variance, suggesting that common method variance was not a substantial concern.

Similarly, the inclusive education orientation scale demonstrated good internal consistency reliability, with a Cronbach's alpha of 0.85 and corrected item–total correlations ranging from 0.52 to 0.71. The KMO value was 0.86, and Bartlett's test of sphericity was significant, χ^2^(28) = 1,198.45, *p* < 0.001, indicating the suitability of the data for factor analysis. Principal axis factoring also supported a unidimensional structure, with the first factor producing an eigenvalue of 4.08 and explaining 51.0% of the total variance. Factor loadings ranged from 0.60 to 0.79. All remaining eigenvalues were below 1.00 (0.86, 0.74, 0.63, 0.51, 0.44, 0.39, and 0.35), supporting the retention of a single-factor solution (Table 2).

To assess the potential influence of common method variance, Harman's single-factor test was conducted using all 16 items from the two study scales. An unrotated exploratory factor analysis identified multiple factors with eigenvalues greater than 1.00, and the first factor accounted for 38.7% of the total variance, which was below the commonly cited 50% threshold. These findings suggest that common method variance was unlikely to fully explain the observed association between teachers' self-efficacy and inclusive education orientations, although shared method effects cannot be entirely ruled out because both constructs were measured using self-report questionnaires.

## Teachers' self-efficacy

Teachers' self-efficacy scores are presented in Table 3. On a 5-point scale, the overall mean score for teachers' self-efficacy was 3.84 (*SD* = 0.62), indicating a moderately high level of perceived self-efficacy in supporting students with ASD in inclusive educational settings. This suggests that participants generally perceived themselves as capable of managing autism-related instructional and classroom demands.

**Table 3:** Teachers' self-efficacy scores (*N* = 381).

Item	Statement	*M*	*SD*
1	I can manage classroom behavior effectively when teaching students with autism.	3.92	0.84
2	I can use a variety of teaching strategies to meet the needs of autistic learners.	4.05	0.78
3	I feel confident in adapting lesson plans to include students with autism.	3.88	0.81
4	I can create a positive learning environment that supports autistic students.	4.12	0.76
5	I feel capable of collaborating with special education staff to support inclusion.	3.74	0.89
6	I can motivate students with autism to participate actively in classroom activities.	3.69	0.91
7	I am confident in assessing the academic progress of autistic learners.	3.80	0.87
8	I can manage stress when teaching students with autism.	3.55	0.95
Total scale score	Teachers' self-efficacy, mean of 8 items	3.84	0.62

Items were rated on a 5-point Likert scale from 1 = strongly disagree to 5 = strongly agree. Higher scores indicate higher perceived self-efficacy.

At the item level, the highest mean scores were observed for creating a positive learning environment that supports autistic students (*M* = 4.12, *SD* = 0.76) and using a variety of teaching strategies to meet the needs of autistic learners (*M* = 4.05, *SD* = 0.78). Comparatively lower scores were found for managing stress when teaching students with autism (*M* = 3.55, *SD* = 0.95) and motivating students with autism to participate actively in classroom activities (*M* = 3.69, *SD* = 0.91). These item-level findings are reported descriptively, while the main interpretation is based on the overall scale score.

## Inclusive education orientations toward students with ASD

Teachers' inclusive education orientation scores are presented in Table 4. The overall mean score was 3.88 (*SD* = 0.59), indicating generally positive self-reported orientations toward inclusive education for students with ASD. This result suggests that participants generally reported favorable beliefs, attitudes, contextual perceptions, and selected reported behaviors related to autism inclusion in mainstream school settings.

**Table 4:** Teachers' inclusive education orientations toward students with ASD (*N* = 381).

Item	Statement	*M*	*SD*
1	Inclusive education benefits both autistic students and their peers.	4.18	0.74
2	I believe students with autism should be taught in mainstream classrooms whenever possible.	3.92	0.81
3	I feel my school provides adequate resources to support inclusion of autistic learners.	3.45	0.97
4	I actively adapt classroom materials for students with autism.	3.78	0.85
5	I believe teacher training on autism is essential for effective inclusion.	4.12	0.77
6	I am open to learning new strategies for teaching students with autism.	4.07	0.79
7	I consider collaboration with parents as an important part of inclusion.	3.84	0.88
8	I believe inclusive education improves social outcomes for autistic students.	3.69	0.92
Total scale score	Inclusive education orientations, mean of 8 items	3.88	0.59

Items were rated on a 5-point Likert scale from 1 = strongly disagree to 5 = strongly agree. Higher scores indicate more positive inclusive education orientations.

At the item level, the highest mean scores were observed for the statements that inclusive education benefits both autistic students and their peers (*M* = 4.18, *SD* = 0.74), teacher training on autism is essential for effective inclusion (*M* = 4.12, *SD* = 0.77), and teachers are open to learning new strategies for teaching students with autism (*M* = 4.07, *SD* = 0.79). Comparatively lower mean scores were observed for perceived adequacy of school resources to support inclusion (*M* = 3.45, *SD* = 0.97) and the belief that inclusive education improves social outcomes for autistic students (*M* = 3.69, *SD* = 0.92).

## Differences in teachers' self-efficacy by selected characteristics

Differences in teachers' self-efficacy according to selected demographic and professional characteristics are presented in Table 5. The difference in self-efficacy scores between male and female teachers was not statistically significant, *t* (379) = −1.92, *p* = 0.056, *d* = 0.20. No statistically significant differences in self-efficacy were found according to educational qualification, *F*_(3, 377)_ = 2.18, *p* = 0.090, η^2^ = 0.017, or school level, *F*_(2, 378)_ = 0.64, *p* = 0.529, η^2^ = 0.003.

**Table 5:** Differences in teachers' self-efficacy by selected demographic and professional characteristics.

Variable	Category	*M*	*SD*	Test statistic	*p*	Effect size
Gender	Male (*n* = 168)	3.79	0.63	T (379) = −1.92	0.056	d = 0.20
	Female (*n* = 213)	3.89	0.61			
Educational qualification	Diploma (*n* = 47)	3.73	0.66	F_(3, 377)_ = 2.18	0.090	η^2^ = 0.017
	Bachelor's degree (*n* = 211)	3.82	0.62			
	Master's degree (*n* = 94)	3.91	0.59			
	PhD (*n* = 29)	3.95	0.56			
Teaching experience	< 5 years (*n* = 88)	3.70	0.65	*F*_(3, 377)_ = 4.23	0.006	η^2^ =0.033
	5–10 years (*n* = 134)	3.88	0.60			
	11–15 years (*n* = 96)	3.91	0.59			
	>15 years (*n* = 63)	3.96	0.57			
Training on inclusion	Yes (*n* = 176)	3.95	0.58	t (379) = 3.12	0.002	*d* = 0.32
	No (*n* = 205)	3.75	0.64			
Type of teacher	General education (*n* = 232)	3.78	0.63	t (379) = 2.32	0.021	d = 0.24
	Special education (*n* = 149)	3.93	0.59			
School level	Primary (*n* = 145)	3.87	0.61	*F*_(2, 378)_ = 0.64	0.529	η^2^ = 0.003
	Intermediate (*n* = 121)	3.81	0.63			
	Secondary (*n* = 115)	3.83	0.62			
Experience teaching students with ASD	Yes (*n* = 192)	3.94	0.58	t (379) = 3.20	0.001	d = 0.33
	No (*n* = 189)	3.74	0.64			

M, mean; SD, standard deviation; ASD, autism spectrum disorder. The Benjamini–Hochberg false discovery rate procedure was applied across the seven group comparison tests reported in this table; the pattern of statistical significance remained unchanged.

A statistically significant difference was found in self-efficacy across teaching experience groups, *F*_(3, 377)_ = 4.23, *p* = 0.006, η^2^ = 0.033. Teachers with more than 15 years of experience reported the highest self-efficacy score, while teachers with less than 5 years of experience reported the lowest score. Bonferroni *post hoc* comparisons showed that teachers with less than 5 years of experience had significantly lower self-efficacy than teachers with more than 15 years of experience, mean difference = −0.26, 95% CI (−0.43, −0.09), *p* =0.004.

Teachers who had received prior training on inclusive education reported significantly higher self-efficacy than those without such training, *t* (379) = 3.12, *p* = 0.002, *d* = 0.32. Special education teachers also reported significantly higher self-efficacy than general education teachers, *t* (379) = 2.32, *p* = 0.021, *d* = 0.24. In addition, teachers with prior experience teaching students with ASD reported higher self-efficacy than those without such experience, *t* (379) = 3.20, *p* = 0.001, *d* = 0.33. After applying the Benjamini–Hochberg false discovery rate adjustment across the seven self-efficacy group comparison tests, the differences by teaching experience, prior training on inclusion, teacher role, and prior experience teaching students with ASD remained statistically significant. Gender, educational qualification, and school level remained non-significant.

## Differences in inclusive education orientations by teacher role and ASD teaching experience

Given the conceptual relevance of teacher role and autism-specific teaching experience in autism inclusion, additional comparisons were conducted for inclusive education orientation scores. Special education teachers reported significantly more positive inclusive education orientations (*M* = 3.97, *SD* = 0.56) than general education teachers (*M* = 3.82, *SD* = 0.60), *t* (379) = 2.43, *p* = 0.016, *d* = 0.25. Teachers with prior experience teaching students with ASD also reported higher inclusive education orientation scores (*M* = 3.98, *SD* = 0.55) than teachers without such experience (*M* = 3.78, *SD* = 0.61), *t* (379) = 3.35, *p* = 0.001, *d* = 0.34.

After applying the Benjamini–Hochberg false discovery rate adjustment across the group comparison tests reported in Tables 5, 6 the significant findings for teaching experience, prior training on inclusion, teacher role, and prior experience teaching students with ASD remained statistically significant. The non-significant findings for gender, educational qualification, and school level remained non-significant.

**Table 6:** Differences in inclusive education orientations by teacher role and ASD teaching experience.

Variable	Category	M	SD	Test statistic	p	Effect size
Type of teacher	General education (*n* = 232)	3.82	0.60	*t* (379) = 2.43	0.016	*d* = 0.25
	Special education (*n* = 149)	3.97	0.56			
Experience teaching students with ASD	Yes (*n* = 192)	3.98	0.55	*t* (379) = 3.35	0.001	*d* = 0.34
	No (*n* = 189)	3.78	0.61			

M, Mean; SD, Standard Deviation; ASD, Autism Spectrum Disorder. The Benjamini–Hochberg false discovery rate procedure was applied across the group comparison tests reported in Tables 5, 6; the pattern of statistical significance remained unchanged.

## Correlation between teachers' self-efficacy and inclusive education orientations

The correlation analysis is presented in Table 7. A statistically significant positive correlation was found between teachers' self-efficacy and inclusive education orientations, *r* = 0.62, *p* < 0.001. This indicates that teachers with higher perceived self-efficacy tended to report more positive orientations toward inclusive education for students with ASD.

**Table 7:** Correlation between teachers' self-efficacy and inclusive education orientations.

Variable	Teachers' self-efficacy	Inclusive education orientations
1. Teachers' self-efficacy	-	
2. Inclusive education orientations	0.62^***^	-

r = Pearson correlation coefficient. ^***^p < 0.0*01*.

However, because both variables were measured using self-report Likert-scale items, this association should be interpreted cautiously. The correlation may reflect a substantive relationship between the constructs, but it may also partly reflect shared method variance and conceptual proximity between teachers' perceived capability and their reported orientation toward inclusion.

## Multiple regression analysis of inclusive education orientations

The results of the multiple regression analysis examining factors associated with inclusive education orientations are presented in Table 8. The overall regression model was statistically significant, *F*_(13, 367)_ = 22.41, *p* < 0.001, and explained 44% of the variance in inclusive education orientations, *R*^2^ = 0.44, adjusted *R*^2^ = 0.42.

**Table 8:** Multiple regression analysis of factors associated with inclusive education orientations.

Predictor variable	*B*	*SE*	β	95% CI for B	*t*	*p*
Teachers' self-efficacy	0.46	0.06	0.48	0.34, 0.58	7.67	< 0.001
Gender, female	0.08	0.05	0.07	−0.02, 0.18	1.58	0.115
Teaching experience, 5–10 years	0.08	0.07	0.06	−0.05, 0.21	1.18	0.239
Teaching experience, 11–15 years	0.11	0.07	0.08	−0.03, 0.25	1.57	0.117
Teaching experience, >15 years	0.17	0.08	0.10	0.01, 0.33	2.15	0.032
Training on inclusion, yes	0.16	0.06	0.14	0.04, 0.28	2.67	0.008
Educational qualification, Bachelor's degree	0.04	0.08	0.03	−0.12, 0.20	0.50	0.617
Educational qualification, Master's degree	0.08	0.09	0.06	−0.10, 0.26	0.89	0.374
Educational qualification, PhD	0.11	0.11	0.05	−0.11, 0.33	1.00	0.318
Type of teacher, special education	0.13	0.06	0.11	0.01, 0.25	2.17	0.031
School level, intermediate	−0.03	0.06	−0.02	−0.15, 0.09	−0.50	0.617
School level, secondary	0.04	0.06	0.03	−0.08, 0.16	0.66	0.511
Prior experience teaching students with ASD, yes	0.14	0.05	0.12	0.04, 0.24	2.80	0.005

Model statistics: F _(13, 367)_ = 22.41, p < 0.001; R^2^ =0.44; adjusted R^2^ =0.42. Reference categories were male, less than 5 years of teaching experience, no prior training on inclusion, diploma qualification, general education teacher, primary school level, and no prior experience teaching students with ASD. CI, confidence interval; B, unstandardized coefficient; SE, standard error; β, standardized coefficient.

Teachers' self-efficacy showed the strongest association with inclusive education orientations, *B* = 0.46, SE = 0.06, β =0.48, *t* = 7.67, *p* < 0.001, 95% CI (0.34, 0.58). This indicates that higher perceived self-efficacy was associated with more positive inclusive education orientations after adjusting for demographic and professional variables.

Prior training on inclusion was also significantly associated with inclusive education orientations, *B* = 0.16, *SE* = 0.06, β =0.14, *t* = 2.67, *p* =0.008, 95% *CI* (0.04, 0.28). Teaching experience of more than 15 years was significantly associated with higher inclusive education orientation scores compared with the reference group of less than 5 years of experience, *B* = 0.17, *SE* = 0.08, β = 0.10, *t* = 2.15, *p* = 0.032, 95% CI (0.01, 0.33). Special education teachers also reported significantly higher inclusive education orientation scores than general education teachers, *B* = 0.13, *SE* = 0.06, β =0.11, *t* = 2.17, *p* = 0.031, 95% CI (0.01, 0.25). Prior experience teaching students with ASD was also significantly associated with more positive inclusive education orientations, *B* = 0.14, *SE* = 0.05, β = 0.12, *t* = 2.80, *p* = 0.005, 95% CI (0.04, 0.24).

Gender, educational qualification, school level, and the lower teaching-experience categories were not statistically significant in the adjusted model.

## Discussion

The present study examined teachers' self-efficacy and its association with self-reported inclusive education orientations toward students with ASD in mainstream schools in Riyadh, Saudi Arabia. Overall, participating teachers reported moderately high self-efficacy and generally positive orientations toward autism inclusion. Teachers' self-efficacy was strongly associated with inclusive education orientations and remained the strongest associated factor in the regression model after adjusting for selected demographic and professional variables. These findings suggest that teachers who perceive themselves as more capable of supporting autistic students also tend to report more positive attitudes, beliefs, contextual perceptions, and selected inclusion-related behaviors.

From a theoretical perspective, this finding is consistent with Bandura's concept of self-efficacy, which emphasizes that individuals' beliefs about their capabilities influence effort, persistence, and performance in demanding situations (Bandura and Wessels, 1997). In autism-inclusive classrooms, teachers may need to adapt instruction, manage communication and behavioral needs, support participation, collaborate with special education staff, and respond to diverse learning profiles. Therefore, teachers' perceived capability is highly relevant to how they approach inclusion. However, because the study was cross-sectional, the association should not be interpreted as evidence that self-efficacy causes more positive inclusive education orientations. It is also possible that successful inclusion-related experiences strengthen teachers' confidence over time through mastery experience (Waddington, 2023). Thus, the findings are best understood as supporting an association between self-efficacy and inclusion-related orientations, with possible reciprocal influences that require longitudinal research to clarify.

The findings also highlight the autism-specific nature of teacher self-efficacy. Teachers reported relatively high confidence in creating a positive learning environment and using varied teaching strategies, but lower confidence in managing stress and motivating students with ASD to participate actively. This pattern suggests that teachers may feel more confident in general instructional planning than in the emotional, behavioral, and engagement-related demands of autism inclusion. Autism-inclusive classrooms often require sustained attention to communication differences, social participation, sensory needs, transitions, and individualized supports. Therefore, teacher preparation should move beyond broad inclusion awareness and focus more directly on practical autism-specific competencies.

The significant association between prior training and more positive inclusive education orientations supports the importance of structured professional development. Training may improve teachers' confidence by increasing autism-related knowledge, reducing uncertainty, and providing practical strategies for adapting instruction and managing classroom challenges. This interpretation is consistent with (Johnson et al. 2021), who emphasized the role of professional development in strengthening teachers' confidence when working with students with ASD. In the Saudi mainstream school context, such training should be continuous rather than limited to short awareness sessions. It should include classroom-based coaching, behavior support strategies, differentiated instruction, family collaboration, and consultation with special education professionals.

Teaching experience was also associated with inclusive education orientations, particularly among teachers with longer professional experience. This finding may reflect the role of mastery experiences in developing confidence and more positive inclusion-related beliefs. Teachers with more years of experience may have had more opportunities to encounter diverse learners, adjust classroom strategies, and develop practical judgment. This aligns with Martins and Chacon (2021), who identified teacher education and professional experience as important sources of teacher self-efficacy. However, experience alone may not be sufficient unless it includes meaningful exposure to autistic students and supportive school conditions. Repeated experience without adequate resources, training, or collaboration may not necessarily lead to stronger efficacy.

The comparison between general education and special education teachers is important because these groups may differ in preparation, role expectations, and exposure to students with ASD. Special education teachers reported higher self-efficacy and more positive inclusive education orientations than general education teachers. This may reflect their more specialized training and greater familiarity with disability-related instructional and behavioral support. However, because inclusive education occurs within mainstream classrooms, general education teachers remain central to successful autism inclusion. These findings suggest that professional development should not be limited to special education teachers. General education teachers also need systematic preparation, access to consultation, and practical support to implement autism-inclusive strategies confidently.

Prior experience teaching students with ASD was also associated with higher self-efficacy and more positive inclusive education orientations. This finding supports the view that autism-specific exposure may be particularly important for developing teacher confidence. Teachers who have directly worked with autistic students may better understand the practical demands of inclusion, including communication support, classroom participation, behavioral regulation, and individualized adaptation. However, such experience should be supported by structured mentoring and school-level resources to prevent stress and burnout. Without adequate support, direct exposure may increase pressure rather than improve confidence.

One important finding was the relatively lower rating for perceived adequacy of school resources. This suggests that participating teachers may generally support inclusion and report positive orientations toward students with ASD, while still perceiving institutional barriers that limit implementation. This distinction is important because positive attitudes and self-efficacy alone cannot guarantee effective inclusion if schools lack specialized support, teaching materials, classroom accommodations, and collaborative structures. In Saudi Arabia, where autism services and inclusive education are still developing, this issue is particularly relevant (Al-Dakroury et al., 2022). The findings therefore suggest that improving autism inclusion requires both teacher-level and school-level interventions.

The study contributes to the literature in several ways. First, it provides context-specific evidence from participating teachers in Riyadh, Saudi Arabia, where empirical work on teachers' self-efficacy and autism inclusion in mainstream schools remains limited. Second, it supports the argument that autism inclusion should be examined separately from general inclusion because autistic students may present distinct instructional, communication, sensory, and behavioral support needs. Third, the study clarifies that teachers' inclusive education orientations are associated not only with self-efficacy, but also with training, teaching role, experience, and prior exposure to students with ASD. However, because convenience sampling was used and a complete sampling frame for all eligible teachers was not available, the findings should not be generalized to all teachers in Riyadh or Saudi Arabia without caution.

The findings should also be interpreted cautiously because both main constructs were measured using self-report questionnaire items. Harman's single-factor test indicated that the first unrotated factor explained 38.7% of the total variance, suggesting that common method variance was unlikely to fully account for the observed association between teachers' self-efficacy and inclusive education orientations. Nevertheless, shared method effects cannot be completely excluded because both constructs were measured using the same response format and data collection method. Therefore, the results should be interpreted as reflecting teachers' perceived capability and self-reported orientations toward autism inclusion, rather than direct evidence of observed classroom practice.

In practical terms, the findings suggest that teacher education and professional development in Saudi mainstream schools should prioritize autism-specific inclusion skills. Training programs should include practical modules on differentiated instruction, visual supports, structured classroom routines, communication support, positive behavior strategies, sensory-aware classroom management, and strategies to increase participation among autistic students. School leaders should also provide ongoing mentoring, collaboration time between general and special education teachers, access to specialists, and adequate classroom resources. These supports may help teachers apply autism-inclusive strategies more confidently and consistently.

Finally, future research should build on these findings using designs that can examine change over time and provide stronger evidence about directionality. Longitudinal studies could clarify whether self-efficacy predicts later inclusion-related orientations or whether successful inclusive experiences strengthen self-efficacy. Intervention studies could evaluate whether autism-specific professional development improves teacher confidence and reported or observed inclusive practices. Future studies should also include classroom observation, multi-informant data, and larger probability-based samples across different regions of Saudi Arabia. Such approaches would help determine whether teachers' self-reported orientations correspond to actual inclusive classroom implementation.

## Conclusion

This study found that teachers in Riyadh reported moderately high levels of self-efficacy and generally positive self-reported inclusive education orientations toward students with autism. Teachers' self-efficacy showed a strong positive association with inclusive education orientations and remained the most prominent associated factor in the regression model. In addition, teaching experience, prior training on inclusion, teaching role, and prior experience teaching students with autism were associated with more positive inclusion-related orientations, whereas gender and educational qualification were not significant in the adjusted model.

These findings suggest that teacher self-efficacy is an important professional factor in autism inclusion within mainstream school settings. They also indicate that practical experience, autism-specific exposure, and targeted training may play meaningful roles in strengthening teachers' readiness to support students with autism in inclusive classrooms. However, the findings should be interpreted within the limits of the study design. Because the study was cross-sectional and relied on self-reported questionnaire data, the results support associations rather than causal relationships and should not be interpreted as direct evidence of observed classroom practice.

Overall, the study contributes context-specific evidence from Saudi Arabia and emphasizes the importance of strengthening teacher preparation, autism-specific professional development, and school-level support for inclusive education. Further longitudinal, intervention-based, and observational research is needed to clarify how teacher self-efficacy develops over time and how it can be enhanced to support more effective inclusion of students with autism in mainstream schools.

## Limitations

Several limitations should be considered when interpreting the findings of this study. First, the study used a cross-sectional design, which limits the ability to determine causal relationships or the temporal direction of associations between teachers' self-efficacy and inclusive education orientations. Accordingly, the findings should be interpreted as evidence of association rather than causation.

Second, the study relied on a convenience sampling strategy. Therefore, the sample may not be representative of all teachers involved in autism inclusion in Riyadh or Saudi Arabia. Although both general education and special education teachers were recruited, the only accessible official population figure available for recruitment planning referred to special education teachers in Riyadh. This figure was used as a pragmatic reference only and should not be interpreted as providing a probability-based sample size calculation, confidence interval, or margin of error for the mixed sample of general and special education teachers. This mismatch between the available reference population and the recruited sample represents a methodological limitation and should be considered when interpreting the findings.

Third, the data were collected using a self-administered questionnaire. Therefore, the results may have been influenced by self-report bias, including social desirability and subjective overestimation or underestimation of teachers' confidence and inclusion-related orientations. Relatedly, the study assessed teachers' reported attitudes, beliefs, contextual perceptions, and selected reported behaviors rather than direct classroom observations. Therefore, the findings should not be interpreted as evidence of actual inclusive classroom practice.

Fourth, because both main constructs were measured using the same self-report questionnaire format, common method variance may have influenced the observed association between teachers' self-efficacy and inclusive education orientations. Harman's single-factor test suggested that common method variance was unlikely to fully account for the findings. However, this test is only a basic diagnostic procedure and cannot completely rule out shared method effects. Future studies should use stronger approaches, such as multi-informant data, temporal separation of measurements, marker variables, or confirmatory factor analytic techniques, to assess and reduce common method bias more rigorously.

Fifth, although the Benjamini–Hochberg false discovery rate procedure was applied to address multiple group comparisons, the inferential findings should still be interpreted cautiously. The group comparison analyses were exploratory and were conducted to identify patterns across selected demographic and professional characteristics. Therefore, emphasis should be placed on the consistency, direction, and effect sizes of the findings rather than statistical significance alone.

Sixth, although the questionnaire was reviewed by experts, pilot-tested, and demonstrated acceptable psychometric properties in the present sample, further validation is still needed. Formal measurement invariance testing between the Arabic and English versions was not conducted. Therefore, although translation and back-translation procedures were used, equivalence between the two language versions cannot be fully confirmed. Future studies should conduct confirmatory factor analysis and measurement invariance testing to strengthen evidence for the instrument's validity across language groups.

Seventh, the inclusive education orientation scale included attitudes, beliefs, contextual perceptions, and selected reported behaviors. Although these components were treated as a combined construct in the present study, they are not identical concepts. In particular, perceived school resources represent a contextual factor rather than a teacher attitude or behavior. Future research may benefit from separating these components into clearer subdomains, such as attitudes toward inclusion, reported inclusive practices, perceived school support, and collaboration.

Eighth, responses from teachers with and without prior experience teaching students with autism were analyzed within the same overall dataset. Although prior ASD teaching experience was examined as a relevant professional variable, teachers without direct experience may have interpreted some autism-specific items differently from those with direct classroom exposure. Future studies should consider stratified analyses or separate subgroup analyses based on direct ASD teaching experience.

Finally, the study was conducted in Riyadh, Saudi Arabia, and the findings may reflect contextual features specific to this educational setting. Therefore, caution is needed when extending the results to other regions or educational systems. Future studies may benefit from probability-based sampling, multi-region recruitment, longitudinal or intervention designs, classroom observation, multi-informant data, and more advanced psychometric testing to strengthen the robustness and generalizability of the findings.

## Data Availability

The raw data supporting the conclusions of this article will be made available by the authors, without undue reservation.
